# Psychometric validation of Chinese version of the immunotherapy of the M.D. Anderson Symptom Inventory for Early-Phase Trials module in a multi-cancer cohort

**DOI:** 10.3389/fimmu.2026.1722663

**Published:** 2026-03-31

**Authors:** Tiantian Fan, Liangzheng Wu, Zhuoma Lacuo, Yujing Liu, Xiaodan Wu

**Affiliations:** 1 Guangzhou Institute of Cancer Research, The Affiliated Cancer Hospital, Guangzhou Medical University, Guangzhou, Guangdong, China; 2 School of Nursing, Sun Yat-sen University, Guangzhou, Guangdong, China; 3 Department of Gastrointestinal Surgery, The Seventh Affiliated Hospital of Sun Yat-sen University, Guangzhou, Guangdong, China; 4 Department of Gastric Surgery, State Key Laboratory of Oncology in South China, Guangdong Provincial Clinical Research Center for Cancer, Sun Yat-sen University Cancer Center, Guangzhou, Guangdong, China; 5 Unit of Psychiatry, Department of Public Health and Medicinal Administration, & Institute of Translational Medicine, Faculty of Health Sciences, University of Macau, Macao, Macao SAR, China

**Keywords:** immune-related adverse events, immunotherapy, MDASI, MDASI-immunotherapy EPT, validation

## Abstract

**Objective:**

The Immunotherapy of the M.D. Anderson Symptom Inventory for Early-Phase Trials module (MDASI-Immunotherapy EPT) was initially developed to assess the severity of symptoms in tumor patients undergoing immunotherapy. However, in the application of this scale, it was observed that the scale did not cover the wide range of symptoms patients reported. Therefore, the scale was revised to reflect such symptoms more comprehensively based on previous studies and expert advice.

**Methods:**

A comprehensive approach was employed to identify symptoms associated with immunotherapy, encompassing a systematic literature review, semi-structured interviews with clinicians and patients, Delphi methodology, and cognitive interviews. Based on item analysis and assessments of reliability and validity, 15 immunotherapy-specific items were ultimately selected for inclusion in the revised MDASI scale.

**Results:**

Through systematic literature review, semi-structured interviews, Delphi consensus, and cognitive interviews, 17 new immunotherapy-specific symptoms were identified. Following item analysis in Study 2.1 (n=396), 9 items were excluded, resulting in a final 34-item scale comprising 13 core symptoms, 15 immunotherapy-specific items, and 6 interference items. Exploratory factor analysis in an independent sample (Study 2.2, n=418) revealed a 6-factor structure (skin symptoms, digestive system symptoms, cardiac symptoms, hepatobiliary system symptoms, extremity edema and musculoskeletal symptoms, pain and fever dimensions) explaining 77.59% of the total variance. The revised MDASI-Immunotherapy EPT demonstrated excellent internal consistency reliability, with Cronbach’s alpha values of 0.917 for the core subscale, 0.878 for the immunotherapy module, and 0.910 for the interference subscale. Criterion validity analysis using Spearman’s correlations revealed significant associations with FACT-G physical well-being domain (ρ = 0.551-0.674, p < 0.01). Subgroup analyses confirmed consistent psychometric properties across urban and rural populations and across major cancer types.

**Conclusions:**

The modified MDASI-Immunotherapy EPT is a valid, reliable, and sensitive tool for measuring symptomatic toxicity in patients receiving immunotherapy.

## 1 Introduction

Immunotherapies are arguably the most important development in the treatment of cancer over the last decade. Expanding indications for these agents have reshaped many standard treatments and have given new hope to patients with cancer (1). One of the costs of these advances has been the emergence of a new set of immune-related adverse events (irAEs) (2), which are often distinct from traditional chemotherapy-related toxicities. The incidence of these adverse events is as high as 70% in patients treated with PD-1/PD-L1 antibodies (3). Adverse reactions of any grade can include fatigue, loss of appetite, skin reactions, endocrine disruption, arthralgia, fever, and drug-induced hepatitis (4). Although most adverse reactions can be managed by appropriate treatment, these symptoms undoubtedly add to the treatment burden and psychosocial stress of patients (5).

Standardized assessment tools are necessary and important in order to better manage the associated symptoms. Among patient self-reported tools, the M.D. Anderson Symptom Inventory (MDASI) stands out for its ability to measure a broad spectrum of symptoms and their interference with daily activities. The development of the Immunotherapy Module for Early-Phase Trials (MDASI-Immunotherapy EPT) specifically addresses the unique symptomatic toxicities associated with immunotherapy (6). The MDASI-Immunotherapy EPT has been translated into Chinese in order to render the assessment culturally and linguistically appropriate for Chinese cancer patients (7). However, the psychometric properties of the MDASI have only been demonstrated in Chinese colorectal cancer patients. It remains unclear whether these findings can be generalized to other types of cancer. Our study will evaluate the applicability and psychometric properties of the MDASI in a wider range of tumor patients.

In practice, however, when the scale was applied, it became apparent that the scale did not fully capture the range of symptoms that patients reported (8). The literature search was necessary according to the criteria, and qualitative interviews should be conducted with patients receiving immunotherapy to make certain that the revised MDASIs were appropriate for patients receiving immunotherapy, that content areas were comprehensive, and the items can be easily understood by the patients. This study will further validate the reliability and validity of the scale in a broader and more diverse patient population including multiple disease types.

## 2 Methods

### 2.1 Study design

**Figure 1:**
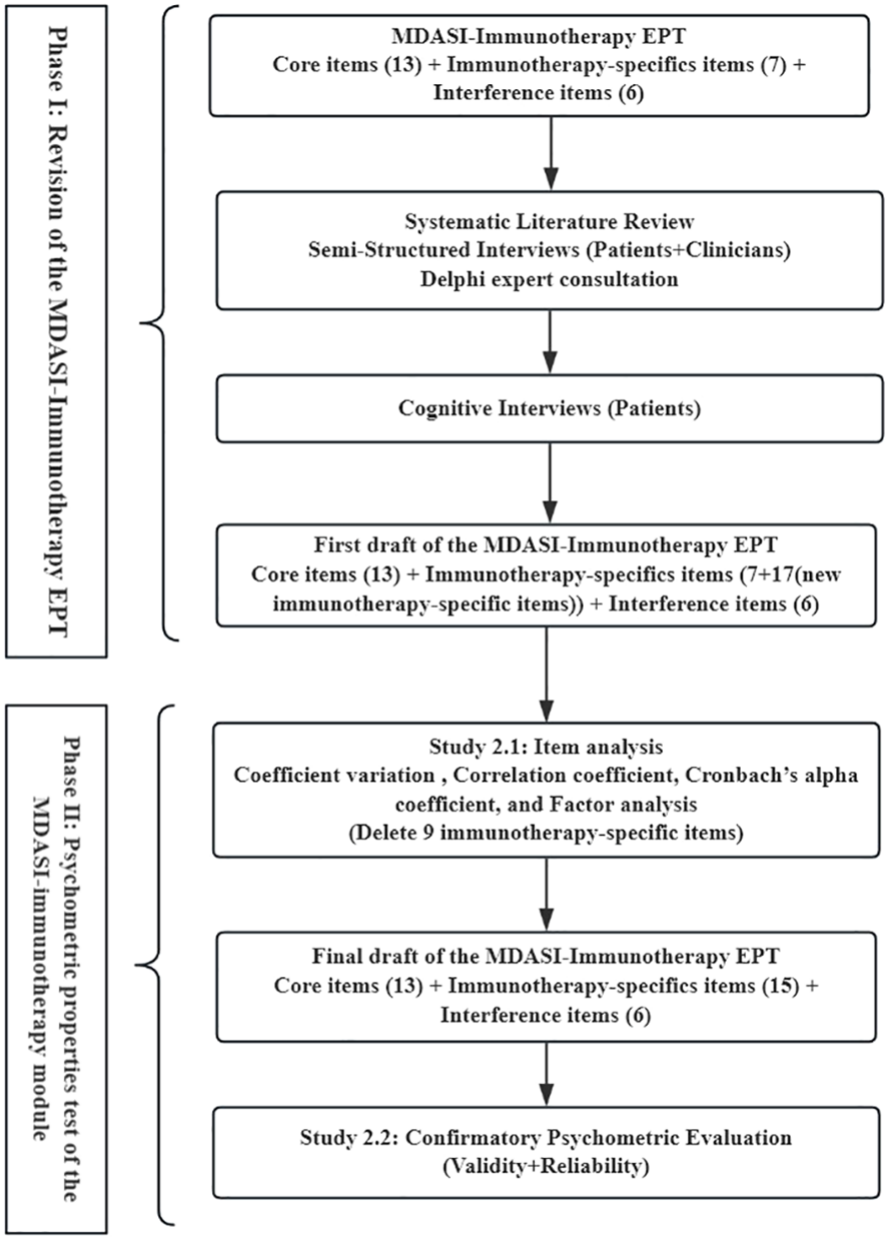
Flowchart.

This study employed a sequential two-phase mixed-methods approach. Phase I focused on revising the MDASI-Immunotherapy EPT to form the preliminary version of MDASI—immunotherapy module. Phase II consisted of two quantitative studies: Study 2.1 item analysis and Study 2.2 psychometric assessment. The study flowchart is presented in **Figure 1**.

#### 2.1.1 Phase I: revision of the MDASI-immunotherapy EPT

##### 2.1.1.1 Systematic literature review

A structured literature review was conducted to identify immunotherapy-related symptoms reported in previous studies. We searched electronic databases including PubMed, Embase, Web of Science, Cochrane Library, China National Knowledge Internet and Wanfang, screening relevant literature published from the inception of each database up to March 2022. Search terms included: “immune checkpoint inhibitors”, “PD-1/PD-L1 inhibitors”, “immunotherapy adverse events”, “patient-reported symptoms”, “symptom burden”, and “quality of life”. Inclusion criteria were: (1) studies involving cancer patients receiving immunotherapy; (2) reporting patient-reported symptoms or quality of life; (3) published in English or Chinese.

##### 2.1.1.2 Semi-structured interviews

We conducted semi-structured interviews with two stakeholder groups between April–July 2022:

Patients (n=20): Purposively sampled from oncology clinics to ensure diversity in cancer type, age, and treatment duration. Inclusion criteria: age ≥18 years, receiving PD-1/PD-L1 inhibitor therapy.

Clinicians (n=10): Participants included medical oncologists (n=6) and oncology nurses (n=4), all of whom possessed over two years’ experience in immunotherapy.

Interview guides covered: (1) symptom experiences during immunotherapy; (2) symptom impact on daily function; (3) gaps in existing assessment tools. Interviews continued until thematic saturation was reached. All interviews were audio-recorded, transcribed verbatim, and analyzed using thematic analysis (9). Symptoms mentioned by ≥20% of participants were retained for further evaluation.

##### 2.1.1.3 Delphi expert consensus process

To systematically evaluate the relevance and clinical importance of the candidate symptom items, a formal two-round Delphi consensus procedure was conducted. Using purposive sampling, 11 multidisciplinary experts were selected based on the following inclusion criteria: (1) holding a postgraduate degree or above, with a senior or associate senior professional title in their respective fields; (2) possessing a minimum of 5 years of specialized experience in oncology clinical practice, immunotherapy management, or symptom research; (3) being actively involved in patient care, clinical research, or the development of patient-reported outcome measures related to immunotherapy; (4) demonstrating a high level of commitment and willingness to participate in the full consultation process.

##### 2.1.1.4 Cognitive interviews

To ensure clarity, comprehensibility, and completeness of the drafted items, cognitive interviews were conducted with 10 patients receiving immunotherapy. Participants were asked to complete the preliminary MDASI-Immunotherapy module and provide feedback on item wording, response options, and overall relevance. Interviews were analyzed to identify ambiguous or difficult items, which were then revised iteratively.

#### 2.1.2 Phase II: Psychometric properties test of the MDASI—immunotherapy module

##### 2.1.2.1 Study 2.1: item analysis

###### 2.1.2.1.1 Participants and procedures

In study 2.1, the preliminary version of the MDASI for immunotherapy included 43 items, based on 5 to 10 subjects for each variable (10), a minimum sample size of 215 was estimated, and taking into account the 20.0% loss rate, at least 269 participants were required.

Participants were recruited between December 2022 and September 2023, which were recruited from two cancer centers in Guangzhou and one general hospital in Shenzhen from China. The hospitals involved in the study are reference centers for the treatment of cancer patients in China, admitting patients from every provinces from whole China. Therefore, these patients are a representative sample. The inclusion criteria were: (1)histologically confirmed cancer; (2)age ≥18 years; (3)receiving PD-1/PD-L1 inhibitor therapy; (4)providing informed consent. The sample size meets statistical requirements.

###### 2.1.2.1.2 Item analysis

The 24 items (original 7 items plus new 17 immunotherapy-specific items) of the preliminary version of the MDASI—immunotherapy module were screened by using the following methods: coefficient variation (CV), correlation coefficient, Cronbach’s alpha coefficient, and factor analysis. The screening criteria were as follows: (a) items with CV values <25% were deleted, (b) correlation between the item score and the scale score was calculated. Items with a correlation coefficient <0.3 or P >0.05 were deleted, (c) if Cronbach’s alpha coefficient increased after an item was deleted, it was considered for deletion. (d) in the exploratory factor analysis (EFA), items with factor loading value <0.4, aggregated into a single dimension, with cross-loaded on multiple dimensions, or inconsistent with theoretical dimensions were considered for deletion. If there or more screening results suggest that an item should be retained, it was retained; otherwise, it was deleted.

##### 2.1.2.2 Study 2.2: psychometric assessment

###### 2.1.2.2.1 Participants and procedures

An independent sample of 418 patients was recruited between September 2023 and June 2024 from the same participating centers, applying the identical eligibility criteria described in Study 2.1: (1) histologically confirmed cancer diagnosis; (2) age ≥18 years; (3) currently receiving PD-1/PD-L1 inhibitor therapy and having completed at least one treatment cycle; (4) providing written informed consent. Patients were excluded if they had cognitive impairment, were concurrently participating in other interventional trials, or had a life expectancy of less than three months. Based on the final 34-item scale, the minimum required sample size was estimated using the recommended ratio of 5 to 10 participants per item for factor analysis (11), with an additional 20% allowance for potential attrition, yielding a target of at least 213 participants. The achieved sample of 418 substantially exceeds this requirement, providing robust statistical power for the validation analyses.

###### 2.1.2.2.2 Measures

In addition to the preliminary version of the MDASI for immunotherapy, a structured self-report were used to collected sociodemographic information and a clinical characteristics information, included age, sex, education, marital status, cancer site, cancer stage, and prior treatment et.al.

The Functional Assessment of Cancer Therapy General (FACT-G) is a cross-culturally acceptable cancer-specific health-related quality of life (HRQL) questionnaire that comprises four subscales: Physical Well-being, Social & Family Well-being, Emotional Well-being, and Functional Well-being (12). The Chinese version of the Functional Assessment Cancer Therapy General (FACT-G) has high pertinences and credibility (13). The scale uses the Likert 5-level scoring method, and converted to standardized score of hundred-mark system for statistical evaluation, with higher scores indicating worse quality of life for patients. In this study, Cronbach’s alpha for the FACT-G was 0.874.

Eastern Cooperative Oncology Group (ECOG)

Eastern Cooperative Oncology Group (ECOG) performance status (PS) was used to describe the patient’s level of functioning (14). ECOG PS is a physician-­rated measure of functional ability using scores ranging from 0 (fully active; able to carry on all predisease performance without restrictions) to 4 (completely disabled; cannot perform self-care; totally confined to bed or chair).

###### 2.1.2.2.3 Validity

The content validity of the MDASI—immunotherapy module was evaluated using the results of the second round of expert consultation (Item-Content Validity Index (I-CVI)>0.78, Scale Content Validity Index (S-CVI)>0.90. The EFA was performed to determine the construct validity of the scale (15). The EFA was conducted using Maximum Likelihood analysis (maximum variance orthogonal rotation). To confirm that data were suitable for factor analysis, Kaiser-Meyer-Olkin test (KMO)>0.50 and Bartlett’s test of sphericity were used. Factor retention was determined based on eigenvalues >1, the scree plot, and a cumulative variance explained >50%. Items were considered for exclusion if they met any of the following criteria: (a) factor loading <0.40; (b) communality <0.30; or (c) presence of significant cross-loadings, where the difference between loadings on two factors was less than 0.20 (11). Criterion validity was evaluated by examining the correlations between MDASI-Immunotherapy module scores and FACT-G scores, which served as the external criterion. Spearman’s ρ was used due to the non-normal distribution of symptom data (Shapiro-Wilk test, p< 0.05 for all symptom subscales).

Known-Groups Validity: Known-groups validity was examined by comparing MDASI-Immunotherapy module scores between patients with different levels of functional status as measured by the ECOG PS. Patients were dichotomized into two groups: those with good performance status (ECOG = 0, fully active) and those with reduced performance status (ECOG≥1, restricted in physically strenuous activity or ambulatory but able to carry out light work). Due to the non-normal distribution of the data, the Mann-Whitney U test (non-parametric) was employed to compare mean ranks between the two groups.

###### 2.1.2.2.4 Reliability

The internal consistency reliability of the MDASI for immunotherapy was assessed using Cronbach’s *alpha*, and split-half reliability coefficient. It is generally considered acceptable when Cronbach’s *alpha* and split-half reliability coefficient are > 0.7, and 0.80 or more is recommended (16). In addition, to determine test-retest reliability, 67 patients treated with immunotherapy were surveyed twice with an interval of one week during the same treatment cycle. To ensure that the observed stability reflected measurement reliability rather than clinical changes, only patients who did not receive any new treatments, dose modifications, or interventions between the two assessments were included in this analysis. The Pearson correlation coefficient was used to calculate the correlation between the two surveys.

###### 2.1.2.2.5 Subgroup analyses

To evaluate the consistency of psychometric properties across different populations, participants were stratified by residency status (urban vs. rural) and cancer type (colorectal, gastric, esophageal, and lung). Reliability was assessed using Cronbach’s alpha for each subgroup. The Mann-Whitney U test compared symptom severity between urban and rural patients. The Kruskal-Wallis test examined differences across cancer types, with *post-hoc* comparisons where appropriate. Chi-square tests compared item endorsement rates across cancer types.

### 2.2 Data analysis

Data were analyzed using IBM SPSS Statistics (version 25.0; IBM Corp, Armonk, NY, USA). Continuous variables are presented as mean ± standard deviation, and categorical variables are summarized as frequency (percentage). A two-tailed p-value of < 0.05 was considered statistically significant for all inferential analyses.

## 3 Results

### 3.1 Participant characteristics

In Phase I, we conducted semi-structured interviews with 20 patients receiving immunotherapy and 10 clinicians. The mean age of patients was 42.85 years (SD = 6.81), with 55% being male. Cancer types included gastric cancer (25%), lung cancer (20%), melanoma and liver cancer (15% each), esophageal cancer and colorectal cancer (10% each), and renal cell carcinoma (5%).

In Phase II, 396 patients were recruited in the study 2.1, 418 patients were recruited in the study 2.2, the sociodemographic and clinical characteristics of the participants in the two studies are shown in **Table 1**.

**Table 1:** Sociodemographic and clinical characteristics of participants(N=814).

Characteristics	Study 1 n (%)	Study 2 n (%)
	N=396	N=418
Age (years, Mean ± SD)		
	49.22 ± 12.76	49.34 ± 12.85
Gender		
Male	214 (54.04)	222 (53.11)
Female	182 (45.96)	196 (46.88)
Educational level		
Primary school or less	80 (20.20)	88 (21.05)
Junior high school	91 (22.98)	105 (25.11)
High school	72 (18.18)	75 (17.94)
College or higher	153 (38.64)	150 (35.88)
Marital status		
Married/Cohabited	338 (85.35)	361 (86.36)
Divorce/Separated/Widowed/Single	58 (14.65)	57 (13.64)
Residency		
Rural (Guangdong province)	94 (23.74)	234 (55.98)
Urban (Guangdong province)	217 (54.80)	96 (22.96)
Rural (Non-Guangdong province)	24 (6.06)	61 (14.59)
Urban (Non-Guangdong province)	61 (15.40)	27 (06.45)
Employment		
Full-time (continue to work before illness)	96 (24.24)	76 (18.18)
Full-time (change of employment after illness)	10 (2.53)	17 (04.06)
Demission	96 (24.24)	110 (26.31)
Part-time job	32 (8.08)	34 (08.13)
Other job	162 (40.91)	181 (43.30)
Average monthly income per capital (RMB)		
<3000	108 (27.27)	119 (28.50)
3000~6000	141 (35.61)	144 (34.40)
6000~10000	76 (19.19)	88 (21.10)
10000~15000	30 (7.58)	27 (6.50)
>15000	41 (10.35)	40 (9.60)
Live alone		
Yes	12 (3.03)	14 (3.35)
No	384 (96.97)	404 (96.65)
Type of Pathology		
Colorectal Cancer	176 (44.44)	202 (48.33)
Gastric Cancer	84 (21.21)	76 (18.18)
Lung Cancer	53 (13.38)	59 (14.11)
Esophagus Cancer	83 (20.96)	81 (19.38)
Stage of cancer		
II	30 (7.58)	30 (7.18)
III	153 (38.64)	144 (34.45)
IV	213 (53.78)	244 (58.37)
ECOG score		
0	193 (48.74)	276 (66.03)
1	203 (51.26)	142 (33.97)
Type of ICIs		
PD-1	229 (57.83)	321 (76.79)
PD-L1	167 (42.17)	97 (23.21)

ECOG, Eastern Cooperative Oncology Group; ICIs, Immune checkpoint inhibitors; PD-1, Programmed cell death protein 1; PD-L1, Programmed death-ligand 1.

#### 3.1.1 Phase I results

##### 3.1.1.1 Literature review findings

The systematic review identified multiple symptoms not fully captured in the original MDASI−Immunotherapy EPT. These included dermatologic symptoms such as pruritus and skin dryness (17);gastrointestinal-related weight loss; pulmonary symptoms including cough (7); musculoskeletal manifestations like stiffness; systemic/constitutional symptoms such as fatigue (18); and symptoms related to other organ systems including palpitations, tachycardia (cardiac), jaundice (hepatic), and oliguria (renal). These symptoms were consistently reported across multiple cancer types and align with documented immune-related adverse events described in the literature.

##### 3.1.1.2 Qualitative interview findings

Thematic analysis of patient and clinician interviews revealed 17 additional immunotherapy-specific symptoms. Respiratory symptoms (cough, dyspnea) and cardiovascular sensations (palpitations, tachycardia) were frequently reported by patients but often under-recognized in clinical assessments. Dermatological symptoms (rash, pruritus, dry skin) and systemic symptoms (fever, fatigue) were confirmed as highly prevalent.

##### 3.1.1.3 Delphi expert consensus results

Eleven multidisciplinary experts participated in two rounds of Delphi consultation. The response rate was 100% for both rounds. In the first round, expert feedback led to specific revisions of item descriptors. The wording for “Myalgia” and “Chest pain” was clarified and refined to enhance clinical specificity and patient comprehension. Based on these revisions, the expert panel demonstrated high authority in the second round, with a judgment coefficient (Ca) of 0.964, a familiarity index (Cs) of 0.945, and a comprehensive authority coefficient (Cr) of 0.955. The Kendall’s coefficient of concordance (W) for the final ratings was 0.377 (p<0.001), indicating a statistically significant consensus among the experts after the iterative revision process.

##### 3.1.1.4 Cognitive interview outcomes

All 10 patients demonstrated clear understanding of the 17 new items. Minor wording adjustments were made to three items based on patient feedback. No items were reported as ambiguous or irrelevant.

Based on the integration of findings from the literature review, qualitative interviews, Delphi process, and cognitive debriefing, a revised MDASI-Immunotherapy module was developed. The first draft of the MDASI-Immunotherapy module comprised 43 items: 13 core MDASI symptoms, 7 original immunotherapy items, 17 new immunotherapy items, and 6 interference items.

#### 3.1.2 Phase II results: psychometric assessment

##### 3.1.2.1 Item analysis

24 immunotherapy-specific items had the CV values > 25% in coefficient variation and all items with a correlation coefficient |*r*|> 0.3 and *P* < 0.05. Moreover, when either item was deleted, the Cronbach’s *alpha* decreased. Furthermore, 4 items were not consistent with the theory, 1 item aggregated into a single dimension, and 4 item cross-loaded on two dimensions, all of 9 items were excluded in the EFA. Therefore, combining the results of the 4 methods, a total of 9 immunotherapy-specific items were deleted (**Table 2**).

**Table 2:** Items analysis results of MDASI-immunotherapy module (N = 396).

Immunotherapy-specific items	Coefficient variation (CV)	Correlation coefficient (r)	Cronbach’s alpha	Factor loading	Result
Rash	95.07%	0.464***	0.926	0.885	retain
Diarrhea	114.74%	0.464***	0.924	0.875	retain
Pain in the abdomen	121.31%	0.677***	0.923	0.686	retain
Swelling of hands, legs, or feet	185.00%	0.605***	0.923	0.751	retain
Headache	125.47%	0.696***	0.922	0.760	retain
Night sweats	185.00%	0.669***	0.922	0.485	retain
Fever and/or chills	150.17%	0.698***	0.922	0.720	retain
Myalgia/Arthralgia	112.91%	0.580***	0.925	0.857^c^	delete
Cough	144.74%	0.657***	0.922	0.601^a^	delete
Dry skin	97.20%	0.657***	0.924	0.682	retain
Weight decreased	122.38%	0.720***	0.922	0.652	retain
Dyspnea	140.03%	0.656***	0.922	0.589^a^	delete
Pruritus	90.30%	0.481***	0.925	0.893	retain
Skin depigmentation	149.40%	0.449***	0.925	0.698^a^	delete
Chest pain	167.49%	0.735***	0.921	0.716/0.458^b^	delete
Pollakiuria/urgent urination	160.92%	0.720***	0.921	0.554/0.460^b^	delete
Joint swelling	142.36%	0.625***	0.923	0.782^a^	delete
Hyperphagia	130.14%	0.560***	0.923	0.561/0.488^b^	delete
Bloody stool and mucous stool	138.28%	0.472***	0.925	0.501/0.549^b^	delete
Musculoskeletal stiffness	147.36%	0.571***	0.924	0.848	retain
Palpitations	131.54%	0.557***	0.923	0.886	retain
Tachycardia	117.37%	0.551***	0.923	0.895	retain
Skin yellow or eyes yellow or urine yellow	138.31%	0.400***	0.926	0.921	retain
Hypourocrinia	130.98%	0.497***	0.925	0.884	retain

***P<0.001.

a The dimension was not consistent with the theory.

b The symptom was cross-loaded on two dimensions.

c The dimensions only included one symptom.

##### 3.1.2.2 Validity

###### 3.1.2.2.1 Content validity

The content validity of the MDASI—immunotherapy module was evaluated using the results of the second round of expert consultation. The Item-Content Validity Index (I-CVI) of the MDASI—immunotherapy module ranged from 0.82 to 1.00 (> 0.78) and Scale Content Validity Index (S-CVI) for the overall module was 0.98 (> 0.90).

###### 3.1.2.2.2 Construct validity

After item analysis, the EFA was performed on the 15 items (original 7 items plus new 8 immunotherapy-specific items) of the final version of the MDASI—immunotherapy module. The KMO value was 0.823 and the Bartlett’s test of Sphericity reached statistical significance (*χ^2^* = 3172.344, *P* < 0.001), confirming the data’s suitability for factor analysis. Following the Kaiser criterion (eigenvalues > 1) and inspection of the scree plot, we fixed the number of factors to 6 (skin symptoms, digestive system symptoms, cardiac symptoms, hepatobiliary system symptoms, extremity edema and musculoskeletal symptoms, pain and fever dimensions), which explained 77.59% of the total variance. As shown in **Table 3**, all items exhibited strong factor loadings ranging from 0.537 to 0.913 on their respective dimensions, with no significant cross-loadings observed.

**Table 3:** Construct validity of the M. D. Anderson Symptom Inventory—immunotherapy module (N = 418).

Symptom	Factor 1	Factor 2	Factor 3	Factor 4	Factor 5	Factor 6
Rash	0.913					
Dry skin	0.554					
Pruritus	0.888					
Pain in the abdomen		0.643				
Diarrhea		0.881				
Weight decreased		0.705				
Palpitations			0.886			
Tachycardia			0.895			
Skin yellow or eyes yellow or urine yellow				0.909		
Hypourocrinia				0.868		
Swelling of hands, legs, or feet					0.807	
Musculoskeletal stiffness					0.753	
Night sweats						0.537
Headache						0.724
Fever and/or chills						0.600

KMO=0.823.

Spaces indicate that the loading of symptoms on the factor dimension is less than 0.40.

###### 3.1.2.2.3 Criteria validity

Considering the non-normal distribution of the symptom reporting data (as confirmed by Shapiro-Wilk tests, P<0.05 for all subscales), Spearman’s rank correlation coefficients (ρ) were employed to assess criterion validity. This was achieved by examining the relationships between the MDASI-Immunotherapy module scores and the Chinese version of the FACT-G total and subscale scores. The correlation coefficients are shown in **Table 4**. Overall, the results revealed a pattern of associations consistent with the conceptual distinction between symptom burden and health-related quality of life. Regarding the correlations with the FACT-G total score, the core subscale (ρ=0.256, P<0.01), immunotherapy module (ρ=0.217, P<0.01), interference subscale (ρ=0.214, P<0.01), and total scale scores (ρ=0.255, P<0.01) all demonstrated significant positive correlations.

**Table 4:** Correlation of the FACT-G scores with the MDASI—immunotherapy module scores (n = 418).

MDASI—immunotherapy module scores	Physical health	Social/family health	Emotional health	Functional health	FACT-G total
core subscale (13 items)	0.652**	0.051	0.271**	-0.102*	0.256**
immunotherapy module (15 items)	0.551**	-0.021	0.202**	-0.092*	0.217**
interference subscale (6 items)	0.656**	0.008	0.339**	-0.136*	0.214**
total scale scores	0.674**	0.035	0.312**	-0.108*	0.278**
skin symptoms	0.287^**^	0.056	0.135^**^	0.012	0.158^**^
digestive system symptoms	0.592^**^	0.092	0.259^**^	-0.004	0.325^**^
cardiac symptoms	0.240^**^	-0.099^*^	0.105^*^	-0.137^**^	-0.005
extremity edema and musculoskeletal symptoms	0.365^**^	-0.134^**^	0.062	-0.203^**^	-0.007
pain and fever dimensions	0.452^**^	-0.006	0.200^**^	-0.115^*^	0.161^**^
hepatobiliary system symptoms	0.216^**^	-0.085	0.035	-0.193^**^	-0.043

**P<0.01,*P<0.1.

###### 3.1.2.2.4 Known-groups validity

The Mann-Whitney U test was conducted to evaluate whether MDASI-Immunotherapy module scores differed significantly between patients with good (ECOG 0, n=276) and reduced (ECOG ≥1, n=142) performance status. The results are presented in **Table 5**.

**Table 5:** Known-groups validity: Mann-Whitney U test comparing.

Subscale	ECOG 0 (n=276) mean rank	ECOG ≥1 (n=142) mean rank	Mann-whitney U	Z	Asymptotic sig. (2-tailed)
Core subscale (13 items)	204.26	219.68	18150.00	-1.236	0.216
Immunotherapy module (15 items)	205.88	216.54	18597.00	-0.854	0.393
Interference subscale (6 items)	202.87	222.39	17765.00	-1.572	0.116
Total scale scores	204.05	220.09	18092.50	-1.285	0.199

MDASI-Immunotherapy Module Scores by ECOG Performance Status (n=418).

Although none of the comparisons reached statistical significance at the conventional p < 0.05 level, the consistent pattern of higher mean ranks in the ECOG ≥1 group across all subscales provides directional support for the scale’s known-groups validity.

##### 3.1.2.3 Reliability

The MDASI for immunotherapy subscales demonstrated good internal consistency reliability. The Cronbach’s *alpha* values was 0.917 for the core subscale, 0.878 for the immunotherapy module, and 0.910 for the interference subscale. The Spearman-Brown coefficient values was 0.872 for the core subscale, 0.750 for the immunotherapy module, and 0.915 for the interference subscale. All dimensions as well as the entire immunotherapy module showed good test-retest reliability. Besides, the reliability of each dimension of the MDASI—immunotherapy module **Table 6**.

**Table 6:** Reliability of the MDASI—immunotherapy module (n = 418).

Immunotherapy-specific subscales	Study 2 (n = 418)	Study 2 (n =418)	Test-retest reliability (n = 117)
	Cronbach’s *alpha*	Spearman-brown coefficient (SB)	*R*
Skin symptoms	0.797	0.757	0.993**
Digestive system symptoms	0.743	0.727	0.988**
Hepatobiliary system symptoms	0.869	0.870	0.991**
extremity edema and musculoskeletal symptoms	0.764	0.765	0.979**
Pain and fever dimensions	0.716	0.757	0.987**
Cardiac symptoms	0.930	0.931	0.985**
Entire immunotherapy module	0.878	0.750	0.992**

**P<0.01

##### 3.1.2.4 Subgroup analyses by residency status and cancer type

###### 3.1.2.4.1 Residency status

Cronbach’s alpha coefficients were generally consistent between urban (n=295) and rural (n=123) subgroups across most dimensions (**Table 7**). Rural patients reported significantly higher symptom burden across all subscales (Mann-Whitney U, all p<0.05), though reliability estimates remained comparable between groups.

**Table 7:** Comparison of psychometric properties between urban and rural subgroups (study 2.2).

Cronbach’s α	Urban (n=295)	Rural (n=123)	Statistical test result
Skin symptoms dimension	0.829	0.698	-
Gastrointestinal symptoms dimension	0.720	0.703	-
Cardiac symptoms dimension	0.940	0.904	-
Extremity Edema and Musculoskeletal Symptoms	0.751	0.819	-
Fever/pain dimensions	0.638	0.777	-
Urinary/hepatobiliary symptoms dimension	0.889	0.846	-
Mann-Whitney U Test	Mean Rank	Mean Rank	Mann-Whitney U
Core subscale (13 items)	198.70	235.39	14957.50**
Immunotherapy module (15 items)	203.20	224.62	16283.00*
Interference subscale (6 items)	196.53	240.60	14317.50**
total score	199.35	233.83	15149.50**

**P<0.01,*P<0.1.

Cancer Type: Reliability estimates were generally acceptable across all cancer types. Endorsement rates differed significantly for four dimensions (**Table 8**): cardiac symptoms (χ²=12.08, p<0.01), extremity edema/musculoskeletal symptoms (χ²=11.23, p<0.01), fever/pain (χ²=17.64, p<0.001), and urinary/hepatobiliary symptoms (χ²=12.89, p<0.01). Lung cancer patients consistently showed the highest rates, esophageal cancer patients the lowest. Skin and gastrointestinal symptoms showed no significant differences across cancer types (p>0.05), with consistently high endorsement rates (>80%).

**Table 8:** Reliability of MDASI-immunotherapy module by cancer type (study 2.2).

Dimension	Colorectal (n=202)	Esophageal (n=81)	Gastric (n=76)	Lung (n=59)	Statistical test result
Skin symptoms dimension	0.829	0.817	0.698	0.750	-
Gastrointestinal symptoms dimension	0.720	0.810	0.703	0.734	-
Cardiac symptoms dimension	0.940	0.962	0.904	0.900	-
Extremity Edema and Musculoskeletal Symptoms	0.751	0.739	0.819	0.741	-
Fever/pain dimensions	0.638	0.751	0.777	0.742	-
Urinary/hepatobiliary symptoms dimension	0.889	0.921	0.846	0.780	-
Endorsement Rates of Selected Items by Cancer	Type [n (%)]				Chi-square χ²
Skin symptoms dimension	182 (90.1%)	71 (87.7%)	74 (97.4%)	56 (94.9%)	6.42
Gastrointestinal symptoms dimension	180 (89.1%)	66 (81.5%)	67 (88.2%)	54 (91.5%)	4.18
Cardiac symptoms dimension	141 (69.8%)^a^	41 (50.6%)^b^	52 (68.4%)^ab^	44 (74.6%)^a^	12.08**
Extremity Edema and Musculoskeletal Symptoms	121 (59.9%)^a^	34 (42.0%)^b^	46 (60.5%)^ab^	38 (64.4%)^a^	11.23**
Fever/pain dimensions	172 (85.1%)^a^	53 (65.4%)^b^	60 (78.9%)^ab^	53 (89.8%)^a^	17.64**
Urinary/hepatobiliary symptoms dimension	126 (62.4%)^ab^	39 (48.1%)^b^	54 (71.1%)^a^	43 (72.9%)^a^	12.89**

**P<0.01; a,b,Different lowercase superscript letters within the same row indicate significant differences between groups based on the Bonferroni-corrected *post-hoc* test (P < 0.05).

##### 3.1.2.5 Symptoms severity

The overall mean scores for all symptom items (34) and interference items (6) were 2.34 ± 1.43 and 1.79 ± 1.68 (**Table 9**), respectively. The two most severe symptoms in immunotherapy module reported were “Skin symptoms “ (2.87 ± 2.12) and “Digestive system symptoms” (2.57 ± 2.10).

**Table 9:** Descriptive statistics for the severity of the symptom items of the MDAIS-Immunotherapy EPT(N=418).

	Item(n)	Mean Score	SD
MDAIS-Immunotherapy EPT	34	2.34	1.43
core subscale	13	2.90	1.82
Interference subscale	6	1.79	1.68
Entire immunotherapy module	15	2.07	1.36
Skin symptoms	3	2.87	2.12
Digestive system symptoms	3	2.57	2.10
Hepatobiliary system symptoms	2	1.72	2.08
extremity edema and musculoskeletal symptoms	2	1.19	1.57
Pain and fever dimensions	3	1.89	1.70
Cardiac symptoms	2	1.59	1.78

## 4 Discussion

The MDASI-Immunotherapy EPT-C is an extended version of the original MD Anderson Symptom Inventory, adapted for patients treated with immunotherapy. This modified tool covers the unique symptomatology associated with immunotherapy, which enables a better understanding of the nuances of the patients’ experiences. The methods used in our study included expert consultations, item analysis, and exploratory factor analysis. This comprehensive process has enabled the extension of the original seven-item immunotherapy symptom module to an seven plus eight, fifteen total in this paper. Such an extension covers a wide range of symptoms related to immunity and hence is able to catch nuances associated with the state of a patient undergoing treatment. The extended MDASI-EPT shows excellent internal consistency and reliability; it is well applicable for detecting and assessing immune-related adverse events. This revised tool now extends to the clinician a symptom inventory that is even more expanded and timely in the management of immunotherapy-related side effects.

Comparison with previous validation studies reveals both consistency and enhancements in our findings. The original MDASI-Immunotherapy EPT development study reported (6) Cronbach’s alpha values of 0.91 for the core items, 0.92 for the immunotherapy module, and 0.93 for the interference subscale values remarkably similar to those observed in our study (0.917, 0.878, and 0.910, respectively), demonstrating the stability of the MDASI framework across different populations and cultural contexts. The previous Chinese validation in colorectal cancer patients (7) reported a 3-factor structure for the immunotherapy items, whereas our expanded module revealed a more nuanced 6-factor structure. This difference likely reflects the broader symptom coverage in our revised module (15 vs. 7 items) and the more diverse cancer types included in our sample.

The inclusion of symptoms such as dry skin, weight loss, and palpitations in the revised module reflects symptoms that may have been underestimated in previous versions due to differences in study populations and follow-up time limitations. Early clinical trials typically recruit highly selected patients, focusing on monitoring acute dose-limiting toxicities, and often overlook chronic mild symptoms that accumulate over time (19–21). Among all cancer types, most patients report experiencing dry skin symptoms. While this persistent toxicity compromises quality of life, it is often deemed “non-critical” in clinical trials prioritizing immediate safety endpoints. Weight loss emerged as a significant issue across our multi-cancer cohort—particularly prevalent among gastrointestinal patients—a symptom previously attributed to disease progression rather than immunotherapy effects. Palpitations, as an independent cardiac factor, may be underestimated in short-term trial follow-ups and among healthier subjects with fewer cardiovascular risk factors. These findings suggest that symptom relevance evolves with broader real-world populations and longer observation periods, necessitating their inclusion in comprehensive immunotherapy assessment tools.

The six-factor structure identified in our exploratory factor analysis demonstrates strong alignment with clinically recognized organ-system clusters of immune-related adverse events (irAEs) (22). Skin symptoms correspond to dermatologic irAEs (rash, pruritus, dry skin), reflecting T-cell infiltration of the dermo-epidermal junction. Gastrointestinal symptoms align with immune-mediated colitis, representing breakdown of intestinal immune tolerance. Hepatobiliary symptoms capture immune-mediated hepatitis, reflecting T-cell attack on hepatocytes. Cardiac symptoms represent myocarditis and arrhythmias, involving myocardial antigen reactivity. Extremity edema and musculoskeletal symptoms align with rheumatic and vasculitic irAEs such as arthritis and RS3PE syndrome, reflecting synovial inflammation and vascular permeability changes. Pain and fever capture systemic inflammatory responses driven by cytokine-mediated mechanisms. The high variance explained (77.59%) confirms that these symptom clusters are not statistically arbitrary but reflect coherent biological processes. Importantly, this factor structure moves beyond traditional organ-specific toxicity grading toward a more integrated understanding of how immunotherapy reshapes patients’ symptomatic experience, supporting the clinical utility of the MDASI-Immunotherapy module for targeted irAE monitoring and management.

The psychometric properties of the modified MDASI - Immunotherapy EPT were assessed in a variety of validity and reliability measures. The I-CVI for the items were between 0.82 and 1.00, surpassing the accepted critical value of 0.78, thus showing each item did have strong content validity (23). Moreover, the S-CVI for the whole module was 0.98, surpassing the recognized critical value of 0.90 and hence supporting the content validity of the scale. For construct validity, the Revised MDASI - Immunotherapy EPT was strongly associated with the Functional Assessment of Cancer Therapy (FACT) (24), reflecting a high convergent validity. Its internal consistency measured as Cronbach’s alpha was 0.917 for core subscales, thus further confirming the excellent reliability of the scale.

The modest correlations between MDASI-Immunotherapy module scores and FACT-G domains (ρ ranging from 0.145 to 0.674) are consistent with findings from previous symptom assessment tool validation studies. This pattern reflects the conceptual distinction between symptom burden and health-related quality of life (HRQoL). The MDASI captures acute symptom severity over the past 24 hours, whereas the FACT-G assesses broader, more stable aspects of physical, social, emotional, and functional well-being over the past week. These constructs, while related, are theoretically distinct—patients may experience significant symptoms yet maintain certain aspects of quality of life, or conversely, may have low symptom burden but impaired quality of life due to psychological or social factors. Similar modest correlations (ranging from 0.20 to 0.40) between symptom inventories and HRQoL measures have been reported in previous oncology PRO validation studies (25, 26). The strongest correlations in our study were observed with the physical well-being domain (ρ up to 0.674), which is expected as both instruments capture physical symptoms and their impact.

The known-groups validity analysis revealed a consistent pattern of higher symptom severity scores among patients with reduced performance status (ECOG ≥1) compared to those with good performance status (ECOG 0), as evidenced by higher mean ranks across all subscales. Although these differences did not reach statistical significance, the directional consistency aligns with clinical expectations—patients with poorer functional status tend to experience greater symptom burden.

The lack of statistical significance may be attributable to several factors. First, the ECOG groups were imbalanced (276 vs. 142), which may have reduced statistical power to detect small to moderate effects. Second, ECOG performance status is a global, clinician-rated measure of functional impairment that may not capture the full spectrum of symptom severity experienced by immunotherapy patients. It is possible for patients to experience significant symptoms while maintaining relatively good performance status, particularly in the outpatient setting.

Rural patients reported significantly higher symptom burden across all domains, possibly reflecting disparities in healthcare access, health literacy, or socioeconomic factors. Despite these mean differences, reliability estimates remained generally consistent, supporting the scale’s applicability in both populations.

The stability of reliability estimates across major cancer types supports the broad applicability of the MDASI-Immunotherapy module. The significantly higher endorsement of cardiac, fever/pain, and urinary/hepatobiliary symptoms among lung cancer patients may reflect the complex interplay between lung cancer pathophysiology and immunotherapy-related effects, warranting particular clinical attention in this population. The consistent endorsement of skin and gastrointestinal symptoms across all cancer types confirms their universal relevance as core irAE dimensions.

In our study, the overall mean scores for the 34 symptom items and 6 interference items on the MDASI-EPT were (2.34 ± 1.43) and (1.79 ± 1.68), respectively. These values are comparable to those reported in previous studies utilizing the MDASI-EPT, where mean scores for symptom severity and interference were found to be within a similar range. However, the most severe symptoms identified in our cohort were “cutaneous symptoms” (2.87 ± 2.12) and “gastrointestinal symptoms” (2.57 ± 2.10), which differ from findings in prior research. This discrepancy may be attributed to the more comprehensive assessment of skin and gastrointestinal symptoms in our study, allowing for a more detailed evaluation of adverse reactions in patients receiving immunotherapy. This suggests that our approach to symptom evaluation may provide a more nuanced understanding of the symptom burden associated with immunotherapy, highlighting the importance of tailored symptom assessment tools in this context.

The modified MDASI-Immunotherapy EPT offers clinicians a tool better suited to the unique symptomatic profile of immunotherapy patients. This can lead to more personalized patient management plans and potentially improve patient outcomes by timely recognition and management of side effects (27). The tool’s sensitivity to changes in symptom severity could also be pivotal in clinical trials for evaluating the safety and tolerability of new treatments, aligning with the Food and Drug Administration’s guidance on the use of PROs in drug development (28).

### 4.1 Our study had limitations

First, selection bias may exist, as participants were recruited from specialized tertiary cancer centers in urban Guangdong province. While these centers serve patients from across China, the sample may not fully represent patients treated in community hospitals or rural settings with limited access to specialized immunotherapy management. Second, the symptom burden was assessed exclusively through patient-reported questionnaires, which may not fully align with objective clinical measures. This discrepancy could result from subjective biases or variations in symptom interpretation. To address this, future research should validate the scale against objective indicators, such as laboratory test results or imaging findings, to establish a more accurate relationship between reported symptoms and actual disease severity. Third, Common Terminology Criteria for Adverse Events (CTCAE) toxicity grades were not systematically collected as part of this study’s protocol, precluding known-groups validation against this clinically important anchor. Future studies should examine the relationship between MDASI-Immunotherapy EPT scores and clinician-graded adverse events to establish clinically meaningful thresholds for symptom severity that correspond to treatment-modifying toxicity grades. Fourth, although we confirmed the 6-factor structure in an independent sample, confirmatory factor analysis (CFA) was not performed. Future studies should employ CFA in new, larger samples to formally test the hypothesized factor structure.

## 5 Conclusions

We combined the literature review, reports of patient symptoms from qualitative interviews, and healthcare provider ratings to generate a more comprehensive measure of PRO that will be tested for its psychometric properties for use in assessing specific symptom burdens in patients receiving immunotherapy.

## Data Availability

The raw data supporting the conclusions of this article will be made available by the authors, without undue reservation.
